# Nck1 depletion induces activation of the PI3K/Akt pathway by attenuating PTP1B protein expression

**DOI:** 10.1186/s12964-014-0071-9

**Published:** 2014-11-14

**Authors:** Hui Li, Julie Dusseault, Louise Larose

**Affiliations:** Department of Medicine, Polypeptide Laboratory, McGill University and The Research Institute of McGill University Health Centre, Montreal, QC Canada

**Keywords:** Nck1, PTP1B, PI3K/Akt pathway

## Abstract

**Background:**

Activation of the PI3K/Akt pathway mediates crucial cellular functions regulated by receptor tyrosine kinases, such as cell growth, proliferation, survival and metabolism. Previously, we reported that the whole-body knockout of the Src homology domain-containing adaptor protein Nck1 improves overall glucose homeostasis and insulin-induced activation of the PI3K/Akt pathway in liver of obese mice. The aim of the current study is to elucidate the mechanism by which Nck1 depletion regulates hepatic insulin signaling.

**Results:**

Here, we demonstrate that Nck1 regulates the activation of the PI3K/Akt pathway in a protein tyrosine phosphatase 1B (PTP1B)-dependent mechanism. Indeed, depletion of Nck1 by siRNA in HepG2 cells enhances PI3K-dependent basal and growth factor-induced Akt activation. In accordance, primary hepatocytes isolated from *Nck1*^*−/−*^ mice also display enhanced Akt activation in response to insulin. Activation of the PI3K/Akt pathway in Nck1-depleted HepG2 cells relies on higher levels of tyrosine-phosphorylated proteins and correlates with decreased PTP1B levels. Interestingly, Nck1 and PTP1B in cells are found in a common molecular complex and their interaction is dependent on the SH3 domains of Nck1. Finally, Nck1 depletion in HepG2 cells neither affects PTP1B gene transcription nor PTP1B protein stability, suggesting that Nck1 modulates PTP1B expression at the translational level.

**Conclusion:**

Our study provides strong evidence supporting that the adaptor protein Nck1 interacts with PTP1B and also regulates PTP1B expression. In this manner, Nck1 plays a role in regulating the PI3K/Akt pathway.

## Background

The serine/threonine protein kinase Akt plays an essential role in regulating various critical cellular functions, such as cell growth, proliferation, survival and metabolism [1]. Activation of Akt involves PI3K, which converts PIP2 to PIP3 following its interaction with receptor tyrosine kinases (RTKs) or tyrosine-phosphorylated (pY) substrates of RTKs [2]. PIP3 recruits the protein kinase PDK1 and Akt at the plasma membrane where Akt gets activated through phosphorylation within the activation loop at Thr^308^ by PDK1 [3]. However, full activation of Akt requires additional phosphorylation at Ser^473^ by mTORC2 [4].

Insulin signaling is initiated by binding of insulin to the insulin receptor (IR) tyrosine kinase, which results in IR activation through autophosphorylation on tyrosine residues and then tyrosine phosphorylation of insulin receptor substrates (IRSs) such as IRS-1. This leads to activation of the PI3K/Akt pathway via binding of p85, the regulatory subunit of PI3K, to IRS-1 [5]. Numerous studies have demonstrated that impaired insulin signaling is closely associated with insulin resistance concomitant with obesity and type 2 diabetes [6,7]. PTP1B, the prototype of non-receptor tyrosine phosphatase superfamily, has been demonstrated to negatively regulate insulin signaling using whole-body or tissue-specific PTP1B knockout mice [8-12]. At the molecular level, PTP1B counteracts both IR and IRS-1 tyrosine phosphorylation to inhibit insulin signaling [13,14].

In mammals, the Src homology (SH) domain-containing adaptor protein Nck (non-catalytic region of tyrosine kinase) includes two highly identical members, Nck1 and Nck2, which contain exclusively three SH3 domains at the N-terminus and one SH2 domain at the C-terminus [15]. Originally, Nck was associated with actin cytoplasmic reorganization through coupling RTKs or RTK substrates to downstream effectors regulating cytoskeleton dynamics [16]. We previously reported that Nck1 is involved in regulating translation through its interaction with the β subunit of the eukaryotic initiation factor 2 [17] and more recently, it was shown that Nck1 functions in cap homeostasis by assembling the cytoplasmic capping complex [18]. Furthermore, in agreement with its localization at the endoplasmic reticulum (ER) network [19], we demonstrated that Nck1 is also involved in regulating the unfolded protein response (UPR) [19-22], a signaling network initiated to cope with stress induced by accumulation of unfolded or misfolded proteins in the ER [23]. Supporting this, we reported that diet-induced obese *Nck1*^*−/−*^ mice display improved overall glucose homeostasis and enhanced hepatic insulin signaling that correlates with reduced UPR compared to obese *Nck1*^*+/+*^ littermates [24]. Using the human hepatocellular carcinoma cell line HepG2, we also showed that Nck1 depletion by siRNA promotes insulin signaling, as represented by increased levels of pY IRS-1, Akt phosphorylation on Ser^473^, GSK3β phosphorylation on Ser^9^ and glycogen synthesis in response to insulin [24].

In the present study, we investigate the mechanism by which Nck1 depletion regulates hepatic insulin signaling. Interestingly, we found that in addition to promoting insulin signaling, Nck1 depletion by siRNA in HepG2 cells also enhances basal and other growth factor-induced Akt phosphorylation, which correlates with increased global pY protein levels and decreased PTP1B levels. In addition, we demonstrated that Nck1 interacts with PTP1B through its SH3 domains, and modulates PTP1B protein expression likely at the translational level. Overall, we uncover a role for Nck1 in regulating activation of the PI3K/Akt pathway through a PTP1B-dependent mechanism.

## Results

### Nck1 depletion enhances hepatic Akt phosphorylation and downstream signaling

**Figure 1:**
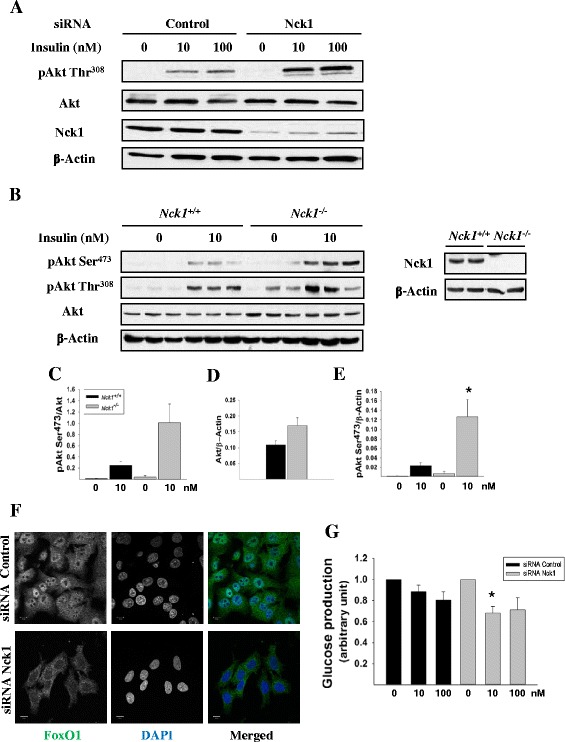
**Insulin-induced Akt phosphorylation and downstream signaling are enhanced in Nck1-depleted cells. (A)** HepG2 cells transfected with control or Nck1 siRNA were exposed to 0, 10 and 100 nM insulin for 5 min after an overnight serum starvation. Equal amount of proteins from total cell lysates were subjected to immunoblot with indicated antibodies. **(B)** Primary hepatocytes isolated from *Nck1* ^*+/+*^ and *Nck1* ^*−/−*^ mice were left untreated or treated with 10 nM insulin for 5 min. Equal amount of proteins from total cell lysates were subjected to immunoblot with indicated antibodies. Bar charts represent mean ± SEM of the ratio of pAkt/total Akt **(C)**, Akt/β-Actin **(D)** or pAkt/β-Actin **(E)** quantified by densitometry analysis. Data are representative of three independent experiments with similar results. *P <0.05 versus *Nck1* ^*+/+*^. **(F)** Representative confocal images of control or Nck1 siRNA-transfected HepG2 cells stained with FoxO1 antibody and DAPI. Images acquired at 63X magnification are representative of three independent experiments with similar results. Scale bars equal 10 μm. **(G)** HepG2 cells transfected with control or Nck1 siRNA were exposed to 0, 10 and 100 nM insulin and analyzed for glucose production by measuring glucose levels in glucose-free DMEM. Glucose production was normalized according to protein contents and expressed as relative value compared to basal. Bar chart represents mean ± SEM from six independent experiments performed in triplicate. *P <0.05 versus siRNA control.

Previously, we reported a role for Nck1 in regulating hepatic insulin signaling both *in vivo* and *in vitro* [24]. In fact, in liver of obese *Nck1*^*−/−*^ mice and in HepG2 cells depleted of Nck1 by siRNA, we observed increased Akt phosphorylation on Ser^473^ in response to insulin compared to controls. Here, we showed that transient transfection of HepG2 cells with Nck1 siRNA, which resulted in more than 90% reduction in Nck1 protein levels, led to enhanced insulin-induced Akt phosphorylation on the activation site Thr^308^ (Figure 1A). To confirm a role for Nck1 in regulating insulin-induced Akt activation in a more physiological setting, primary hepatocytes isolated from normal chow diet (NCD)-fed *Nck1*^*+/+*^ and *Nck1*^*−/−*^ mice were stimulated or not with insulin and total cell lysates assessed for Akt phosphorylation by immunoblot. Interestingly, primary hepatocytes from *Nck1*^−/−^ mice also displayed increased insulin-induced Akt phosphorylation on Ser^473^ and Thr^308^ compared to *Nck1*^*+/+*^ hepatocytes (Figures 1B and C). Although this might be attributed to higher Akt levels in *Nck1*^*−/−*^ hepatocytes (Figure 1D), the absolute levels of phosphorylated Akt (pAkt), the *bona fide* signaling molecule, were significantly increased in *Nck1*^*−/−*^ hepatocytes upon insulin stimulation, as shown by higher pAkt Ser^473^/β-Actin ratio compared to *Nck1*^*+/+*^ hepatocytes (Figure 1E). Together, these data demonstrate that Nck1 is a regulator of insulin-induced Akt phosphorylation.

The transcription factor forkhead box O1 (FoxO1) is known to translocate from the nucleus to the cytosol upon phosphorylation by Akt [25]. To test whether signaling downstream of Akt is upregulated in Nck1-depleted HepG2 cells, we assessed FoxO1 subcellular distribution by immunofluorescence and confocal microscopy. In serum-starved control HepG2 cells, FoxO1 accumulated in the nucleus, whereas nuclear FoxO1 was hardly seen in Nck1-depleted HepG2 cells (Figure 1F), supporting dynamic Akt downstream signaling in these cells. Since phosphorylation of FoxO1 by Akt is well known to limit hepatic glucose production through decreasing transcription of the glucose 6-phosphatase gene [26], we then compared the ability of insulin to inhibit glucose production in HepG2 cells transfected with control or Nck1 siRNA. In accordance with increased Akt activation and cytoplasmic localization of FoxO1, we found that Nck1-depleted HepG2 cells also displayed higher sensitivity toward the inhibitory effect of insulin on glucose production (Figure 1G).

**Figure 2:**
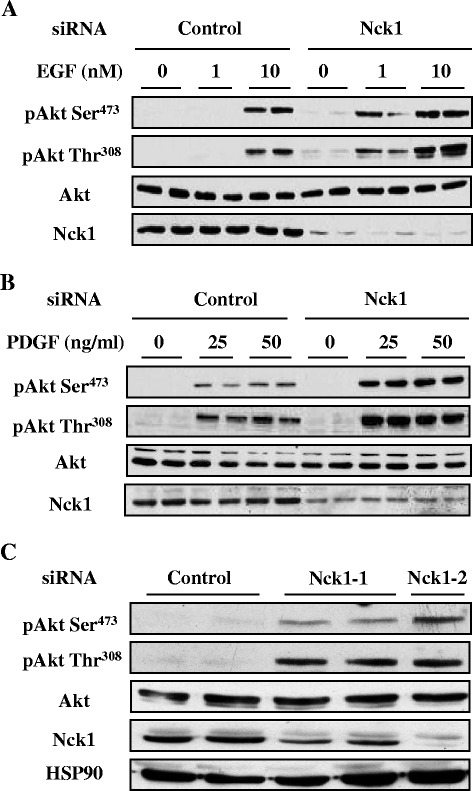
**EGF- and PDGF-induced Akt phosphorylation is enhanced in Nck1-depleted HepG2 cells.** Overnight serum-starved HepG2 cells transfected with control or Nck1 siRNA were exposed to 0, 1 and 10 nM EGF **(A)** or 0, 25 and 50 ng/ml PDGF-BB **(B)** for 5 min. Equal amount of proteins from total cell lysates were subjected to immunoblot with indicated antibodies. Data are representative of three independent experiments performed in duplicate with similar results. **(C)** HepG2 cells transfected with control or different Nck1 siRNAs (Nck1-1, Nck1-2) were starved overnight. Equal amount of proteins from total cell lysates were subjected to immunoblot with indicated antibodies. Data are representative of three independent experiments showing similar results.

To delineate the mechanism by which Nck1 regulates hepatic Akt phosphorylation, we determined whether Nck1 depletion promotes Akt phosphorylation in response to other growth factors. For this, we compared epidermal growth factor (EGF)- and platelet-derived growth factor (PDGF)-induced Akt phosphorylation in HepG2 cells depleted or not of Nck1. In cells transfected with control siRNA, EGF induced Akt phosphorylation on both Ser^473^ and Thr^308^ at 10 nM, while 1 nM of EGF dramatically enhanced Akt phosphorylation on both sites only in Nck1-depleted cells (Figure 2A). Enhanced Akt phosphorylation was also observed in Nck1-depleted cells treated with PDGF (Figure 2B). Interestingly, we constantly observed increased basal Akt phosphorylation in Nck1-depleted cells. To further confirm the negative regulation of Nck1 on basal Akt phosphorylation, we tested two other siRNAs to decrease Nck1 expression in HepG2 cells. As shown in Figure 2C, these two siRNAs, which inhibited Nck1 expression by 62% (Nck1-1) and 94% (Nck1-2) respectively, also enhanced basal Akt phosphorylation on both Ser^473^ and Thr^308^. These findings demonstrate that the effects of Nck1 depletion on promoting Akt phosphorylation are not limited to growth factor-induced Akt phosphorylation, but also apply to basal Akt phosphorylation. Collectively, these results strongly support that Nck1 regulates hepatic Akt activation.

**Figure 3:**
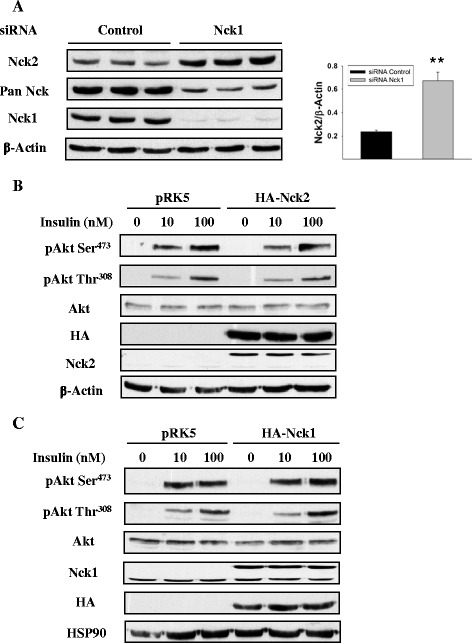
**Akt phosphorylation in HepG2 cells overexpressing Nck. (A)** Total cell lysates from HepG2 cells transfected with control or Nck1 siRNA were subjected to immunoblot with indicated antibodies. Bar charts represent mean ± SEM of the ratio of Nck2/β-Actin. **P <0.01 versus control. Total cell lysates from HepG2 cells transfected with pRK5, HA-Nck2 **(B)** or HA-Nck1 **(C)** plasmids were subjected to immunoblot with indicated antibodies. Data are representative of three independent experiments showing similar results.

Nck protein family includes two members sharing 68% identity, Nck1 and Nck2. Here we showed that HepG2 cells also expressed Nck2 and surprisingly, Nck1 depletion in HepG2 cells resulted in upregulation of Nck2 expression (Figure 3A). To determine whether increased Nck2 levels contributes to the effect of Nck1 depletion on Akt phosphorylation, Nck2 was overexpressed in HepG2 cells and Akt phosphorylation assessed. As shown in Figure 3B, overexpression of Nck2 in HepG2 cells had no effect on basal and insulin-induced phosphorylation of Akt, excluding that elevated Nck2 expression is involved in enhanced phosphorylation of Akt in Nck1-depleted HepG2 cells. On the other hand, overexpression of Nck1 in HepG2 cells also had no effect on Akt phosphorylation (Figure 3C). This may be attributed to the fact that Nck1 is an adaptor protein with various binding partners and its overexpression, by perturbing Nck1 interactome homeostasis, does not essentially lead to the opposite effects of silencing Nck1.

### Enhanced basal Akt phosphorylation in Nck1-depleted HepG2 cells is PI3K-dependent

**Figure 4:**
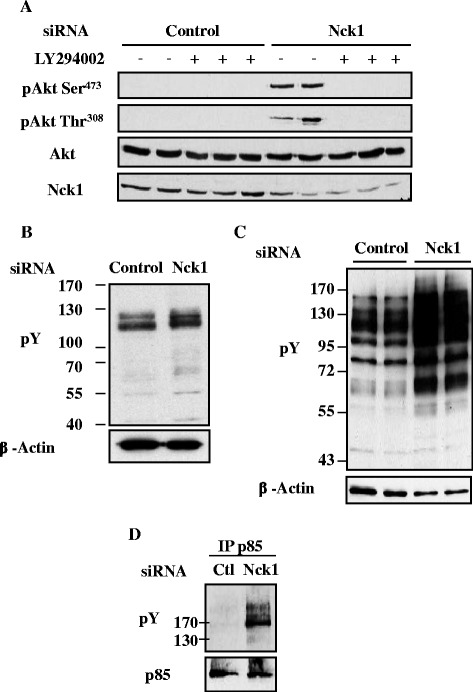
**Enhanced basal Akt phosphorylation in HepG2 cells depleted of Nck1 is dependent on PI3K, and correlates with increased total and p85-associated pY proteins.** Overnight serum-starved HepG2 cells transfected with control or Nck1 siRNA were exposed to 10 μM of the PI3K inhibitor LY294002 for 30 min **(A)**, untreated **(B)** or pretreated with 100 μM of pervanadate (PV) for 5 min **(C)** before harvesting. Equal amount of proteins from total cell lysates were subjected to immunoblot with indicated antibodies. Data are representative of three independent experiments performed in duplicate or triplicate and showing similar results. Total cell lysates prepared from HepG2 cells transiently transfected with control or Nck1 siRNA were subjected to anti-p85 (regulatory subunit of PI3K) immunoprecipitation followed by immunoblot using indicated antibodies **(D)**. Data are representative of three independent experiments showing similar results.

Given that PI3K is an upstream activator of Akt, we assessed whether PI3K mediates the effects of Nck1 depletion on basal Akt phosphorylation. HepG2 cells transfected with control or Nck1 siRNA were treated with the PI3K inhibitor LY294002 and cell lysates subjected to immunoblot to detect Akt phosphorylation. We found that LY294002 completely abolished the elevated basal Akt phosphorylation on Ser^473^ and Thr^308^ in Nck1-depleted cells (Figure 4A), suggesting that activation of PI3K mediates enhanced Akt phosphorylation in these cells. PI3K mainly gets activated following the association of its regulatory subunit p85 with pY proteins, such as activated RTKs and RTK substrates phosphorylated on tyrosine residues [27]. Thus, we analyzed pY protein levels in control and Nck1 siRNA-transfected HepG2 cells pretreated or not with pervanadate (PV), a general tyrosine phosphatase inhibitor [28]. In the absence of pervanadate, pY protein levels were increased in Nck1-depleted cells (Figure 4B) compared to control while PV treatment amplified this difference (Figure 4C). In addition, we observed increased levels of pY proteins associated with the p85 subunit of PI3K in Nck1-depleted cells compared to cells transfected with control siRNA (Figure 4D). Altogether, these data indicate that Nck1 depletion promotes PI3K-dependent Akt phosphorylation in HepG2 cells. Furthermore, this correlates with increased levels of pY proteins and pY proteins associated with p85, suggesting that Nck1 regulates signaling upstream of PI3K.

### Nck1 interacts with PTP1B through its SH3 domains

**Figure 5:**
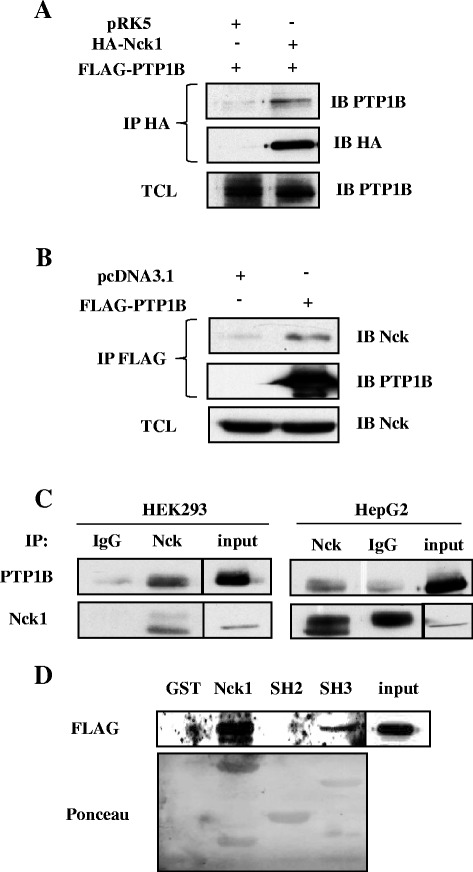
**Nck and PTP1B interaction. (A)** HEK293 cells were transfected with FLAG-PTP1B and pRK5 (empty vector) or HA-Nck1. Total cell lysates were subjected to anti-HA immunoprecipitation followed by immunoblot using indicated antibodies. Data are representative of three independent experiments showing similar results. **(B)** HEK293 cells were transfected with FLAG-PTP1B and pcDNA3.1 (empty vector). Total cell lysates were subjected to anti-FLAG immunoprecipitation followed by immunoblot using indicated antibodies. **(C)** Total cell lysates from HEK293 or HepG2 cells were subjected to IgG or Nck immunoprecipitation followed by immunoblot with indicated antibodies. The vertical line in the immunoblot is to indicate that samples are separated by empty lanes. **(D)** Cell lysates from HEK293 cells overexpressing FLAG-PTP1B were incubated with GST, GST-Nck1, GST-SH2 domain of Nck1, GST-SH3 domains of Nck1 for 3 h followed by immunoblot with anti-FLAG antibody. Ponceau staining reveals the GST-fusion proteins. Data are representative of three independent experiments showing similar results.

PTP1B is known to negatively regulate RTK signaling that leads to activation of the PI3K/Akt pathway. Interaction between PTP61F and Dock, the orthologue of PTP1B and Nck in *Drosophila melanogaster*, was demonstrated using the yeast two-hybrid system and then confirmed by *in vitro* binding assays [29]. In this study, we assessed Nck1/PTP1B interaction in mammalian cells. First, we used HEK293 cells transiently overexpressing HA-Nck1 and FLAG-PTP1B given that compared to HepG2 cells, HEK293 cells can be transfected at higher efficiency. As shown in Figure 5A, in HEK293 cells overexpressing both Nck1 and PTP1B, we detected PTP1B in the HA immunoprecipitates, indicating that Nck1 and PTP1B exist in a common complex when overexpressed. Next, we showed that endogenous Nck coimmunoprecitated with FLAG-PTP1B transiently overexpressed in HEK293 cells (Figure 5B). Finally, in both HEK293 and HepG2 cells, we provided evidence that Nck and PTP1B interacted at the endogenous level (Figure 5C). To identify Nck1 molecular determinant(s) that mediates its interaction with PTP1B, we performed *in vitro* binding assays using bacterial recombinant GST fusion proteins of Nck1 full-length, SH2 or SH3 domains. Lysates prepared from HEK293 cells overexpressing FLAG-PTP1B were incubated with the above GST fusion proteins. As a result, full-length Nck1 and the SH3 domains bind to PTP1B, while the SH2 domain failed to do so (Figure 5D). Overall, these data demonstrate that Nck1 interacts with PTP1B through its SH3 domains in mammalian cells.

### PTP1B expression is downregulated in Nck1-depleted HepG2 cells

**Figure 6:**
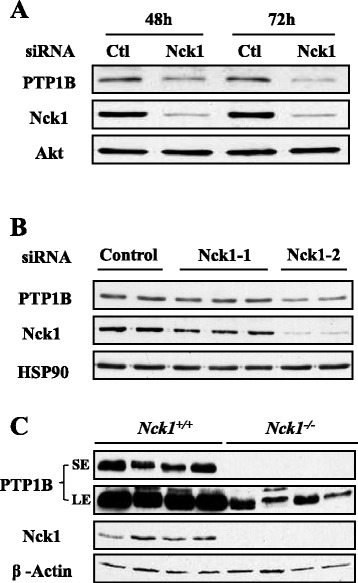
**Nck1 depletion downregulates PTP1B protein expression.** HepG2 cells transfected with control or Nck1 siRNA were harvested at 48 and 72 h **(A)** or 48 h **(B)**. Equal amount of proteins from total cell lysates were subjected to immunoblot with indicated antibodies. Data are representative of three independent experiments showing similar results. **(C)** Liver extracts prepared from *Nck1* ^+/+^ and *Nck1* ^−/−^ mice were subjected to immunoblot with indicated antibodies. Data represent samples of four mice under each genotype. SE: short exposure; LE: long exposure.

**Figure 7:**
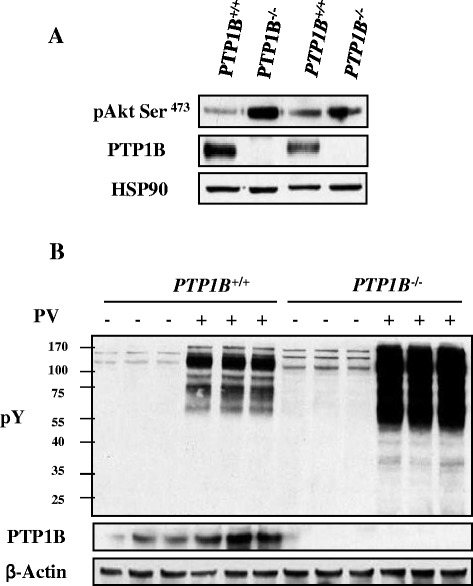
**Genetic deletion of PTP1B mimics the effects of depleting Nck1 on Akt phosphorylation and pY protein levels. (A)** Total cell lysates prepared from *PTP1B* ^+/+^ and *PTP1B* ^−/−^ MEFs were subjected to immunoblot with indicated antibodies. Data are representative of three independent experiments showing similar results. **(B)** *PTP1B* ^+/+^ and *PTP1B* ^−/−^ MEFs were treated with or without 100 μM of PV for 5 min before harvesting. Total cell lysates were subjected to immunoblot with indicated antibodies. Data are representative of three independent experiments performed in triplicate and showing similar results.

**Figure 8:**
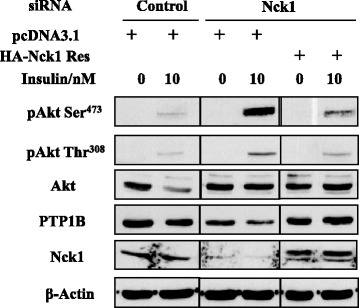
**Reexpression of Nck1 in Nck1-depleted HepG2 cells restores Akt phosphorylation and PTP1B expression.** HepG2 cells expressing pCDNA3.1 or siRNA resistant HA-Nck1 (HA-Nck1 Res) were further subjected to control or siRNA Nck1 transfection. Forty-eight hours after siRNA transfection cells were exposed to 0 and 10 nM insulin for 5 min following an overnight serum starvation. Total cell lysates were subjected to immunoblot with indicated antibodies. The vertical line in the immunoblot is to indicate that samples are separated by extra lanes. Data are representative of three independent experiments showing similar results.

Given that Nck1 interacts with PTP1B, we hypothesized that Nck1 regulates activation of the PI3K/Akt pathway through PTP1B. To test this, we determined PTP1B levels in HepG2 cells transfected with control or Nck1 siRNA. As shown in Figure 6A, PTP1B levels were decreased in HepG2 cells depleted of Nck1. This was confirmed using two other Nck1 siRNAs (Figure 6B). Of note, downregulation of PTP1B levels seemed to correlate well with the extent of Nck1 knockdown. Interestingly, PTP1B levels were robustly decreased in liver of NCD-fed *Nck1*^*−/−*^ mice compared with *Nck1*^*+/+*^ littermates (Figure 6C), strongly supporting our *in vitro* observation. Overall, these data suggest that reduced PTP1B levels contribute to promote activation of the PI3K/Akt pathway in Nck1-depleted cells. Supporting this, we found that Akt phosphorylation was increased in *PTP1B*^*−/−*^ MEFs (Figure 7A). In accordance, total pY protein levels were increased in *PTP1B*^*−/−*^ compared to *PTP1B*^*+/+*^ MEFs, and this difference was even more pronounced in PV-treated cells (Figure 7B). Interestingly, in Nck1 siRNA-transfected HepG2 cells, reintroducing expression of Nck1 by overexpressing a siRNA-resistant form of Nck1 prevented increased phosphorylation of Akt and restored PTP1B levels (Figure 8). Collectively, these results strongly suggest that Nck1 regulates activation of the PI3K/Akt pathway through a PTP1B-dependent mechanism.

**Figure 9:**
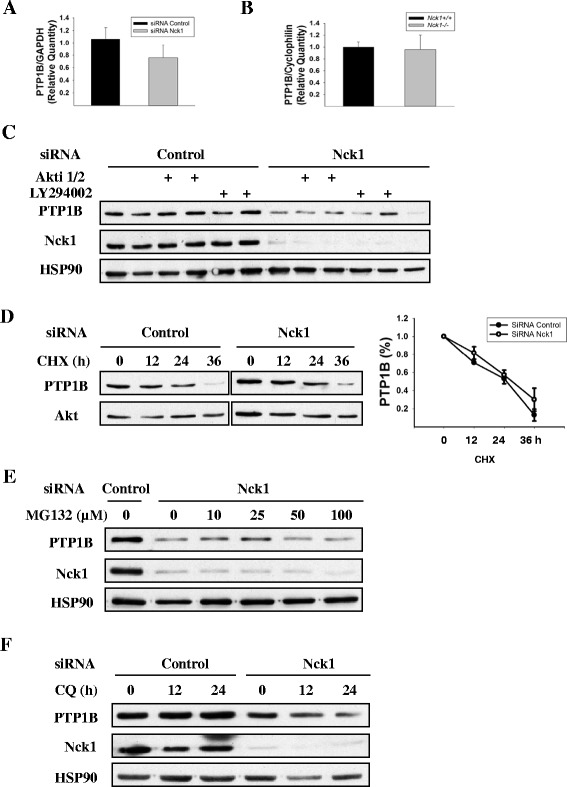
**Nck1 depletion affects neither PTP1B gene transcription nor protein stability. (A)** Quantitative RT-PCR analysis of PTP1B mRNA levels in HepG2 cells transfected with control or Nck1 siRNA. Data are presented as mean ± SEM from five independent experiments. The expression levels of PTP1B mRNA in control siRNA-transfected cells were set to one. **(B)** Quantitative RT-PCR analysis of PTP1B mRNA levels in liver of *Nck1* ^+/+^ and *Nck1* ^−/−^ mice. Data are presented as mean ± SEM of four mice under each genotype. The expression levels of PTP1B mRNA in *Nck1* ^+/+^ mice were set to one. **(C)** HepG2 cells transfected with control or Nck1 siRNA were treated with 10 μM of the PI3K inhibitor LY294002 or 10 μM of the Akt inhibitor Akti 1/2 for 24 h before harvesting. Equal amount of proteins from total cell lysates were subjected to immunoblot with indicated antibodies. Data are representative of three independent experiments performed in duplicate and showing similar results. **(D)** Twelve hours after transfection, HepG2 cells transfected with control or Nck1 siRNA were treated with 100 μg/ml of cycloheximide (CHX) for additional 12, 24 or 36 h. Equal amount of proteins from total cell lysates were subjected to immunoblot with indicated antibodies. The graph represents PTP1B levels at different times of CHX exposure as mean ± SEM from three independent experiments with PTP1B levels at time 0 in control and Nck1 siRNA-transfected cells fixed to one. **(E)** HepG2 cells transfected with control or Nck1 siRNA were treated with 0, 10, 25, 50 and 100 μM MG132 for 6 h. Equal amount of proteins from total cell lysates were subjected to immunoblot with indicated antibodies. Data are representative of three independent experiments showing similar results. **(F)** HepG2 cells transfected with control or Nck1 siRNA were treated with 100 μM chloroquine (CQ) for 12 or 24 h. Equal amount of proteins from total cell lysates were subjected to immunoblot with indicated antibodies. Data are representative of three independent experiments showing similar results.

To investigate the mechanism by which Nck1 depletion decreases PTP1B protein expression, we then compared PTP1B mRNA levels by quantitative real-time PCR (qRT-PCR) on total RNA extracts prepared from HepG2 cells transfected with control or Nck1 siRNA. As shown in Figures 9A, qRT-PCR analyses demonstrated that depletion of Nck1 did not significantly reduce PTP1B mRNA levels. This result was confirmed using liver extracts from *Nck1*^+/+^ and *Nck1*^−/−^ mice (Figure 9B). Therefore, altered PTP1B gene transcription is not involved in reducing PTP1B protein expression in Nck1-depleted HepG2 cells and liver of *Nck1*^−/−^ mice.

Previously, it was shown that Akt phosphorylates PTP1B on Ser^50^ and impairs its ability to dephosphorylate the IR [30]. To ensure that increased Akt activation does not affect PTP1B expression in Nck1-depleted cells, HepG2 cells transfected with control or Nck1 siRNA were treated with the PI3K inhibitor LY294002 or specific Akt inhibitor Akti 1/2 to block Akt activity and cell lysates subjected to immunoblot to detect PTP1B expression. Inhibiting PI3K or Akt did not restore PTP1B expression in Nck1-depleted cells (Figure 9C), suggesting that decreased PTP1B levels in the absence of Nck1 is independent of activation of the PI3K/Akt pathway.

Next, we examined whether depleting Nck1 in HepG2 cells alters PTP1B protein stability. Using cycloheximide, a compound that blocks translation elongation [31], we found that PTP1B protein turnover rate remained the same after depleting Nck1 (Figure 9D), suggesting that protein degradation is not involved in PTP1B downregulation induced by Nck1 depletion. Indeed, neither blocking proteasome-dependent protein degradation by MG132 nor blocking lysosome-dependent protein degradation by chloroquine restored PTP1B expression in Nck1-depleted cells (Figures 9E and F), confirming that reduced PTP1B expression in Nck1-depleted cells is not due to protein degradation. Overall, these data suggest that Nck1 regulates PTP1B protein expression at the translational level.

## Discussion

Previously, we reported that the whole-body *Nck1* knockout improves overall glucose homeostasis in obese mice, in agreement with enhanced hepatic insulin signaling [24]. Besides, we uncovered that siRNA-mediated Nck1 depletion in HepG2 cells also enhances insulin signaling. These data strongly suggest that Nck1 is a regulator of hepatic insulin signaling. However, the mechanism by which Nck1 depletion regulates hepatic insulin signaling remains to be elucidated. In the present study, we report that in addition to insulin-induced Akt phosphorylation, Nck1 depletion also impacts Akt phosphorylation in serum-starved (basal) HepG2 cells and in response to other growth factors such as EGF and PDGF. Furthermore, we demonstrate that elevated Akt phosphorylation in Nck1-depleted HepG2 cells correlates with higher levels of total pY proteins and pY proteins associated with the p85 subunit of PI3K. Meanwhile, we find that Nck1 interacts with PTP1B and regulates its protein expression. Taken together, these data let us propose that Nck1 depletion, by reducing PTP1B expression, enhances tyrosine-phosphorylated proteins that trigger PI3K activation, therefore promoting Akt phosphorylation.

PTP1B is a negative regulator of insulin signaling by dephosphorylating IR and IRS proteins [13,32]. In accordance, whole-body *PTP1B* knockout mice are hypersensitive to insulin and resistant to high fat diet (HFD)-induced insulin resistance [8,9]. In cultured primary human skeletal muscle, manipulating PTP1B expression levels inversely modulates insulin-induced Akt phosphorylation, and increased PTP1B expression in skeletal muscle of patients with type 2 diabetes is associated with decreased whole-body insulin sensitivity [33]. Moreover, *PTP1B*^−/−^ MEFs display marked increase in tyrosine-phosphorylated EGFR [34] and PDGFR [34,35]. In this study, we observe lower PTP1B levels in HepG2 cells depleted of Nck1, which we believe contribute to promote protein tyrosine phosphorylation and activation of the PI3K/Akt pathway, as supported by elevated basal pY protein levels and Akt phosphorylation in MEFs lacking PTP1B. However, the underlying PTP1B substrate(s) that regulates Akt phosphorylation in Nck1-depleted cells remain to be determined, although IR, IRS, EGFR and PDGFR are all well-known targets of PTP1B. Considering the localization of PTP1B at the cytoplasmic face of the ER [36], reduced PTP1B levels in Nck1-depleted cells might induce activation of the PI3K/Akt pathway in a ligand-independent manner by promoting phosphorylation of newly synthesized RTKs during their processing, as reported for the insulin receptor precursors at the ER [37].

Localized at the ER, PTP1B has been involved in the regulation of the UPR initiated upon ER stress [38-40]. Interestingly, we previously demonstrated that Nck1 also localizes at the ER and regulates the UPR [19-22,24]. The UPR is mainly composed of three arms triggered by distinct ER transmembrane proteins: inositol-requiring enzyme 1α (IRE1α), protein kinase R-like ER kinase (PERK) and activating transcription factor 6 (ATF6) [23]. Recently, obesity-induced insulin resistance in peripheral insulin target tissues has been linked to ER stress that results in abnormal activation of the UPR [41-43]. In fact, obesity leads to sustained activation of IRE1α that impairs insulin signaling through IRE1α/JNK-mediated phosphorylation of IRS-1 that prevents IRS-1 tyrosine phosphorylation by activated IR [42]. Whole-body *Nck1* knockout mice are resistant to HFD-induced insulin resistance and ER stress, resembling the phenotype of liver-specific *PTP1B* knockout mice [11,24,44]. Given that Nck1 interacts with PTP1B and modulates PTP1B protein expression as we demonstrate here, it is plausible that the protective effect of Nck1 deficiency against HFD-induced insulin resistance and ER stress could be attributed to PTP1B downregulation in liver of *Nck1*^−/−^ mice.

The interaction between PTP1B and Nck was first reported in *Drosophila melanogaster*, and suggested to involve the SH3 domains of Dock, the Nck orthologue [29,45,46]. This is consistent with the fact that the C-terminus of PTP1B harbors two proline-rich sequences that match the consensus SH3 domain-binding motif [47,48]. In mammalian cells, Nck1 associates with PTP1B independent of insulin stimulation, suggesting a SH3-mediated interaction as well [49]. In this study, we provide clear evidence showing that Nck1 interacts with PTP1B through its SH3 domains. Further experiments are required to characterize which SH3 domain(s) is responsible for this interaction.

In the current study, we demonstrate that PTP1B protein levels are drastically decreased in Nck1-depleted HepG2 cells and liver of *Nck1*^*−/−*^ mice. Interestingly, a recent study reports that *PTP1B*^−/−^ mice fed a NCD or HFD, both display significantly lower levels of Nck1 in skeletal muscle, although the underlying mechanism has not been investigated [50]. Therefore, a reciprocal regulation between Nck1 and PTP1B may exist. Our findings suggest that Nck1 modulates PTP1B expression at the translational level. In fact, qRT-PCR analysis reveals no change of PTP1B gene transcription in HepG2 cells depleted of Nck1 or in *Nck1*^*−/−*^ liver. Using the proteasome inhibitor MG132 or lysosome inhibitor chloroquine, we rule out an effect of Nck1 depletion on PTP1B protein degradation. Furthermore, PTP1B is known to be cleaved at the C-terminal ER-targeting domain by the cysteine protease calpain, releasing a 42 kDa soluble form of PTP1B in the cytosol [51]. Decrease in PTP1B protein levels in Nck1-depleted cells is not attributed to the C-terminal cleavage of PTP1B, since the anti-PTP1B antibody used in this study is raised against its N-terminus, therefore would detect the cleaved form of PTP1B. Taken together, it is then possible that Nck1 affects PTP1B mRNA translation. In agreement with our previous observation that Nck1 modulates protein translation [20], we observed a decrease in general protein synthesis in HepG2 cells depleted of Nck1 as measured by ^35^S-Met/Cyst incorporation (unpublished data). However, given that numerous protein levels (e.g. Akt, β-actin, HSP90), as determined by immunoblot, are not affected in the Nck1-depleted HepG2 cells, Nck1 might play an essential role in maintaining proper translation of specific mRNAs such as PTP1B mRNA. Considering the essential role of Nck1 in assembling the cytoplasmic capping complex [18], it is plausible that depletion of Nck1 results in accumulation of uncapped PTP1B transcripts that are not efficiently translated. Further investigation is required to test this hypothesis. Alternatively, microRNAs, by binding to their target mRNAs, act as posttranscriptional regulators to repress target mRNA translation [52]. Searching TargetScan database reveals several putative microRNA binding sites on the 3’-UTR of human PTP1B mRNA, including miR-1, miR-206, miR-613, miR-124, miR-506, etc. Interestingly, IRE1α branch of the UPR is involved in decreasing microRNA expression either through the IRE1α endoribonuclease activity [53-55] or in a JNK-dependent manner [56]. Considering that Nck1 depletion attenuates IRE1α/JNK signaling [24], further experiments will be performed to determine whether Nck1 depletion affects PTP1B protein expression by indirectly increasing microRNAs that target PTP1B mRNA.

## Conclusions

Our study highlights the implication of PTP1B in mediating Nck1-dependent regulation of the hepatic PI3K/Akt pathway. Since Akt is a critical node in regulating insulin and growth factor biological actions, our findings contribute to identify Nck1 as a potential target in therapeutic strategies for insulin resistance and cancer. Furthermore, the striking effects of PTP1B on metabolic diseases and cancer in preclinical research make it an attractive drug target. However, the development of PTP1B inhibitors is halted by selectivity and bioavailability of these drugs [57,58]. Given the ability of Nck1 to regulate PTP1B expression, Nck1 could be a substitutive target to bypass the bottleneck of developing PTP1B inhibitors.

## Methods

### Cell culture and treatments

Human hepatocellular carcinoma cells (HepG2) were grown in minimum essential Eagle’s medium (MEM; GIBCO) containing 10% fetal bovine serum (FBS; GIBCO) and 1% antibiotic-antimycotic (anti-anti) solution, which contains 10,000U/ml of penicillin, 10,000 μg/ml of streptomycin, and 25 μg/ml of Fungizone® (GIBCO). Human embryonic kidney (HEK) 293 cells, *PTP1B* wild type (*PTP1B*^+/+^) and knockout (*PTP1B*^−/−^) mouse embryonic fibroblasts (MEFs) were grown in Dulbecco's modified Eagle’s medium (DMEM; GIBCO) containing 10% FBS and 1% anti-anti. Cells were incubated at 37°C in a 5% CO_2_ environment. Small interference (si) RNAs against human Nck1 (Nck1) 5’-AACAUCCAUUACAUCUCCUUUCUCGAA-3’, (Nck1-1) 5’-GGAGAUGUAAUGGAU GUUA-3’, (Nck1-2) 5’-GGCCUUCACUCACUGGAAA-3’ were purchased from Integrated DNA Technologies. Scramble siRNA purchased from Ambion was used as negative control. HepG2 cells were transfected using Lipofectamine RNAiMAX reagent (Invitrogen) according to the manufacturer’s instructions, with a final siRNA concentration of 40 nM. HA-Nck1 and HA-Nck2 overexpression in HepG2 cells were performed using Lipofectamine 2000 according to the manufacturer’s instructions. Re-expression of siRNA resistant HA-Nck1 in Nck1 siRNA-transfected HepG2 cells using Lipofectamine 3000 was performed 24 h prior siRNA transfection according to the manufacturer’s instructions. HEK293 cells were transfected with empty vector, HA-Nck1 and/or FLAG-rat PTP1B using CalPhos™ mammalian transfection kit (Clontech) according to the manufacturer’s instructions. HepG2 cells were starved overnight in MEM containing 0.1% bovine serum albumin (BSA, Invitrogen) after 48 h siRNA transfection, and then incubated with either insulin (Eli Lilly Co., Indianapolis, IN), EGF (Fitzgerald Industries International), PDGF-BB (Roche), pervanadate (Sgima), LY294002 (Calbiochem) or Akt inhibitor 1/2 (Calbiochem) at concentrations indicated in Figure legends. Cycloheximide (Calbiochem), MG132 (Calbiochem) or chloroquine diphosphate salt (Sigma) were added to HepG2 cells after siRNA transfection without starvation at concentrations indicated in Figure legends.

### Mice

*Nck1*^*+/−*^ mice were previously obtained from Dr. Tony Pawson (Toronto, Canada) [59] and used to generate *Nck1*^*+/+*^ and *Nck1*^*−/−*^ littermates. Mice were maintained in an animal facility with fixed 12 h light/dark cycle and free access to water and food. All animal care and handling followed the guidelines of McGill University (protocol #5069) according to the standards defined by the Canadian Council on Animal Care. Only male mice were used in this study.

### Liver extracts and isolated primary hepatocytes

Age-matched mice were used to harvest liver or prepare primary hepatocytes. Mice were euthanized using a CO_2_ chamber, tissues immediately collected, snap-frozen in liquid nitrogen, and kept at −80°C for further processing. To prepare tissue extracts, tissues were homogenized using a Polytron at 20% (w/v) in 5 mM Tris · HCl (pH 7.4), 0.25 M sucrose, 1 mM MgCl_2_, 1 mM dithiothreitol, 10 mM sodium pyrophosphate, 1 mM sodium orthovanadate, 100 mM sodium fluoride, and 17.5 mM β-glycerophosphate supplemented with protease inhibitors. Triton X-100 was added to a final concentration of 1%. Following 10 min incubation at 4°C, samples were centrifuged at 13,000 g for 10 min at 4°C. Supernatants were further centrifuged at 200,000 g for 30 min at 4°C. Final supernatants were subjected to Bio-Rad protein assay (Bio-Rad) for protein quantification and equal amount of protein was used for immunoblot analysis.

Mouse primary hepatocytes were isolated according to Renton et al. [60] with minor modifications. Briefly, anesthetized mice were subjected to *in situ* liver perfusion via a cannula inserted in the inferior vena cava through the heart’s right atrium, while the portal vein was cut. Then, liver was first perfused with heparin solution (10 mM HEPES (pH 7.85), 142 mM NaCl, 6.7 mM KCl, 0.6 mM EGTA and 1.5 U/ml Heparin), followed by collagenase solution (10 mM HEPES (pH 7.85), 142 mM NaCl, 6.7 mM KCl, 12 mM CaCl_2_ · 2H_2_O and 200 U/ml collagenase). Perfused liver was transferred to a petri dish, gently teased apart with forceps, filtered through two layers of gauze, washed twice with MEM containing 10% FBS and antibiotics, and then plated in 6-well plates coated with collagen matrix (Vitrogen-100; Collagen Corp.). Primary hepatocytes were cultured as previously reported [61]. Insulin stimulation, carried out in serum-free media, was evaluated two days after initial plating following the conditions indicated in the Figure legends.

### Antibodies

As previously described, rabbit polyclonal anti-Nck1 antibody was generated using GST-fusion protein encoding human Nck1-specific amino acid sequences between the third SH3 and the SH2 domains (Nck1: QNNPLTSGLEPSPPQCDYIRPSLTGKFAGNP) [24]. Rabbit polyclonal anti-p85 antibody was generated using GST-fusion protein encoding the two SH2 domains of the p85 subunit of PI3K. pAkt (Ser^473^ and Thr^308^), Akt, FoxO1, β-Actin and HSP90 antibodies were purchased from Cell Signaling Technology. pY20 antibody was purchased from Santa Cruz Biotechnology, PTP1B antibody from BD Biosciences, anti-HA matrix from Roche, while FLAG antibodies was from Sigma.

### Cell lysates, immunoblot and immunoprecipitation

Cells were washed twice with ice-cold phosphate-buffered saline (PBS) and detergent-soluble proteins were extracted using lysis buffer (50 mM HEPES (pH 7.5), 150 mM NaCl, 10% glycerol, 1% Triton X-100, 1.5 mM MgCl_2_, 1 mM EGTA, 10 mM sodium pyrophosphate, 100 mM sodium fluoride) supplemented with protease inhibitors. Following centrifugation at 13,300 g for 10 min at 4°C, supernatants were subjected to Bio-Rad protein assay. Total cell lysates normalized for protein were resolved by SDS-PAGE, transferred onto nitrocellulose membrane, and immunoblotted with indicated antibodies. Enhanced chemiluminescence (Amersham GE Healthcare) was used to detect immunoreactive proteins according to the manufacturer’s instructions. For p85 immunoprecipitation, total cell lysates prepared from HepG2 cells were incubated with anti-p85 antibody overnight at 4°C. The next day, protein A-agarose (Santa Cruz Biotechnology) was added to the lysate-antibody mixture and samples were further incubated for 2 h at 4°C. For Nck/PTP1B coimmunoprecipitation, total cell lysates were incubated with either anti-HA, anti-FLAG or anti-Nck antibodies for 2 h or overnight at 4°C. Immunoprecipitated proteins were washed three times with lysis buffer, resuspended in Laemmli buffer, heated at 95°C for 5 min and freezed until analysis by immunoblot as described above.

### Glucose production

HepG2 cells transfected with control or Nck1 siRNA were plated in 12-well plates. After an overnight serum starvation, cells were incubated with insulin for 6 h. Then, media were replaced by 0.4 ml/well of phenol red-free, glucose-free DMEM containing 2 mM sodium pyruvate (GIBCO) and 20 mM lactate (Sigma) in the presence of insulin for another 3 h. Finally, these media were collected and subjected to glucose measurement by the Amplex® Red glucose/glucose oxidase assay kit (Invitrogen). Glucose levels were normalized according to protein contents.

### GST pull-down assay

GST, GST-Nck1 full length, GST-Nck1 SH2 domain and GST-Nck1 SH3 domain proteins induced in bacteria according to classical procedures and purified using glutathione agarose beads were mixed with total cell lysates from HEK293 cells overexpressing FLAG-PTP1B for 3 h at 4°C. Following the incubation, beads were washed with lysis buffer. Proteins were recovered in Laemmli buffer and analyzed by immunoblot.

### Immunofluorescence and confocal microscopy

HepG2 cells were plated on coverslips in 6-well plates and transfected with control or Nck1 siRNA. After an overnight serum starvation, cells were fixed in 4% paraformaldehyde for 15 min. Fixed cells were blocked in PBS containing 5% donkey serum and 0.3% Triton X-100 for 1 h followed by an overnight incubation with anti-FoxO1 antibody at 4°C. The next day, cells were incubated with Alexa Fluor® 488 donkey anti-rabbit IgG (H + L) (Invitrogen) for 1 h at room temperature followed by the nuclear staining with 4’, 6-diamidino-2-phenylindole dihydrochloride (DAPI, Sigma). Coverslips were mounted on glass slides using ProLong® Gold antifade reagent (Invitrogen). Images were captured using a Zeiss LSM 510 confocal microscope.

### RNA extraction and quantitative real-time PCR

Total RNA was extracted from transfected HepG2 cells or liver of *Nck1*^+/+^ and *Nck1*^−/−^ mice using TRIzol reagent (Invitrogen) according to the manufacturer’s instructions. RNA was reverse transcribed to cDNA using High Capacity cDNA Reverse Transcription Kit (Life Technologies™). Human PTP1B gene in HepG2 cells was amplified using Taqman® Fast Advanced Master Mix (Life Technologies™) in the ViiA™ 7 Real-Time PCR system (Life Technologies™), while mouse PTP1B gene in liver extracts was amplified using Power SYBR® Green PCR Master Mix (Life Technologies™). Primer sequences used for mouse PTP1B gene were: forward 5’-TGGCCACAGCAAGAAGAAAA-3’ and reverse 5’-GGAAATGCAGGATCTCTCGA-3’. GAPDH and cyclophilin were used as internal control.

### Statistical analyses

Quantification of immunoreactive signal was performed using the analysis software Quantity One (Bio-Rad) on a GS-800 Calibrated Densitometer (BIO-RAD). Results are presented as means ± SEM. Student’s *t*-test statistical analyses (unpaired) were performed using SigmaPlot Version 12 (Systat Software Inc.). P value <0.05 was considered to be statistically significant.

## Abbreviations

RTKs: Receptor tyrosine kinases
IRS: Insulin receptor substrate
IR: Insulin receptor
ER: Endoplasmic reticulum
FoxO1: Forkhead box O1
EGF: Epidermal growth factor
PDGF: Platelet-derived growth factor
GST: Glutathione S-transferase
IRE1α: Inositol-requiring enzyme 1α
PERK: Protein kinase R-like ER kinase
ATF6: Activating transcription factor 6
JNK: cJun N-terminal kinase
NCD: Normal chow diet
HFD: High fat diet
UTR: Untranslated region
UPR: Unfolded protein response
